# PP67 Cardiovascular Comorbidities In Chronic Myeloid Leukemia Patients In The Brazilian Unified Health System: An Administrative Database Analysis

**DOI:** 10.1017/S0266462325102857

**Published:** 2025-12-29

**Authors:** Daniela Pachito, Mariana Sebastião, Rosemeire Lassabia, Paulo H. R. F. Almeida, Thais Senna, Rodrigo F. Alexandre

## Abstract

**Introduction:**

Second-line treatment for Philadelphia-positive chronic myeloid leukemia (Ph+CML) in the Brazilian Unified Health System (SUS) currently involves the tyrosine kinase inhibitors (TKIs) dasatinib and nilotinib. Choice of TKIs should be made considering existing comorbidities and the risk of adverse events. Our objective was to estimate the proportion of patients with cardiovascular comorbidities receiving TKIs in the SUS.

**Methods:**

A retrospective administrative database analysis was conducted of cases recorded from October 2013 to May 2024. The International Classification of Diseases, 10th Revision codes used for chronic cardiovascular diseases were identified. Two databases of the outpatient information system were analyzed, the oncology database and the individualized outpatient production report, to identify patients receiving dasatinib or nilotinib as a second-line treatment for Ph+CML who had at least one outpatient procedure with an International Classification of Diseases, 10th Revision code of cardiovascular disease. To achieve this, an inner join procedure using the cryptographed identifier was implemented. Cases were characterized by age, sex, disease stage, and geographic region.

**Results:**

A total of 6,556 unique patients receiving second-line treatment for Ph+CML were analyzed. Their mean age was 49.4 (standard deviation 16.2). There was a predominance of males (55.6%) and white (47.6%) people. Cases were concentrated in the southeast (48.4%), the most populous region of the country. Most cases were at the chronic phase (70.8%), followed by the accelerated (17.7%) and blast phases (11.5%). Dasatinib was the most used treatment, representing 52 percent of cases, followed by 40.5 percent using nilotinib. Other regimens included chemotherapy and combined treatments. Patients with cardiovascular disease represented 10.9 percent of the total sample; 9.8 percent of patients treated with dasatinib and 13 percent of patients receiving nilotinib.

**Conclusions:**

Real-world administrative data provided information on patient profile, treatment patterns, and disease severity. Moreover, the proportion of patients with Ph+CML who had cardiovascular comorbidities was estimated at the national level. These elements are necessary to assess unmet needs and treatment patterns not compliant with the recommendations from guidelines, to underpin the design of optimized strategies of care.

